# Ionizing irradiation-induced Fgr in senescent cells mediates fibrosis

**DOI:** 10.1038/s41420-021-00741-4

**Published:** 2021-11-12

**Authors:** Amitava Mukherjee, Michael W. Epperly, Donna Shields, Wen Hou, Renee Fisher, Diala Hamade, Hong Wang, M. Saiful Huq, Riyue Bao, Tracy Tabib, Daisy Monier, Simon Watkins, Michael Calderon, Joel S. Greenberger

**Affiliations:** 1 Department of Radiation Oncology, UPMC Hillman Cancer Center, Pittsburgh, PA USA; 2 Department of Biostatistics, University of Pittsburgh, Pittsburgh, PA USA; 3 Department of Hematology/Oncology, UPMC Hillman Cancer Center, Pittsburgh, PA USA; 4 Department of Rheumatology & Clinical Immunology, University of Pittsburgh, Pittsburgh, PA USA; 5 Department of Cell Biology, University of Pittsburgh, Pittsburgh, PA USA

**Keywords:** Mechanisms of disease, Ageing

## Abstract

The role of cellular senescence in radiation-induced pulmonary fibrosis (RIPF) and the underlying mechanisms are unknown. We isolated radiation-induced senescent tdTOMp16 positive mesenchymal stem cells, established their absence of cell division, then measured levels of irradiation-induced expression of biomarkers of senescence by RNA-seq analysis. We identified a Log2 6.17-fold upregulation of tyrosine kinase Fgr, which was a potent inducer of biomarkers of fibrosis in target cells in non-contact co-cultures. Inhibition of Fgr by shRNA knockdown did not block radiation-induced senescence in vitro; however, both shRNA knockdown, or addition of a specific small-molecule inhibitor of Fgr, TL02-59, abrogated senescent cell induction of profibrotic genes in transwell-separated target cells. Single-cell RNA-seq (scRNAseq) analysis of mouse lungs at day 150 after 20 Gy thoracic irradiation revealed upregulation of Fgr in senescent neutrophils, and macrophages before detection of lung fibrosis. Thus, upregulated Fgr in radiation-induced senescent cells mediates RIPF and is a potential therapeutic target for the prevention of this radiation late effect.

## Introduction

The histopathology of radiation pulmonary fibrosis (RIPF) is similar to that of idiopathic pulmonary fibrosis (IPF), silicosis, and in post-infection conditions [1–4]. Recent reports indicate cellular senescence precedes both idiopathic and radiation-induced pulmonary fibrosis (RIPF) [5–7].

Ionizing irradiation induces senescent cells [6, 8], which display specific physiological properties including cell cycle exit, senescent-associated β-galactosidase (SA-β-GAL) expression, and secretion of senescence-associated secretory phenotype (SASP) proteins [9–12]. Cell cycle arrest in senescent cells is associated with an increase in cyclin-dependent kinase inhibitors p21, and p16 which are components of tumor-suppressor pathways regulated by p53 and the retinoblastoma (pRB) proteins respectively [11]. SASP contains proinflammatory cytokines, matrix-remodeling enzymes, and extracellular vesicles [10–12]. SASP proteins also include chemotactic proteins like Ccl2, Ccl4, and transforming growth factor beta (TGFβ), which is a profibrotic cytokine [12]. The SASPs in radiation-induced senescent (RIS) cells [13] differ from oncogenes [14], chemotherapeutic drugs [15], or telomere shortening [14, 16] induced senescence.

Prior studies with mixed senescent and non-senescent cells have limited strong conclusions about their specific properties [16]. A method by which to purify senescence cells would require the elimination of non-senescent cells in the targeted population [17]. We took advantage of a novel mouse strain, in which senescent cells can be sorted by td-Tomato fluorochrome, linked to induction of senescence biomarker p16 [18]. We used a cell line derived from tdTOMp16+ mouse bone marrow to sort a pure population of RIS cells, which display a unique gene expression profile compared to irradiated non-senescent cells in the same experiments and in non-irradiated control cells. Using RNA-seq analyses, we determined that a specific tyrosine kinase, Fgr, is upregulated in senescent cells. Senescent cell induction of fibrosis biomarkers was blocked by the addition of an inhibitor of Fgr, TL02-59 [19], or by shRNA knockdown of Fgr prior to irradiation. Single-cell RNA-seq (ScRNAseq) analysis of irradiated mouse lungs revealed Fgr upregulation in monocytes, neutrophils, and macrophages at 150 days. Therefore, Fgr mediates senescent cell induction of fibrosis, and its inhibition by TL02-59 provides a novel strategy by which to ameliorate RIPF.

## Results

### Radiation induction and sorting of senescent tdTOMp16+ cells in vitro

**Fig. 1:**
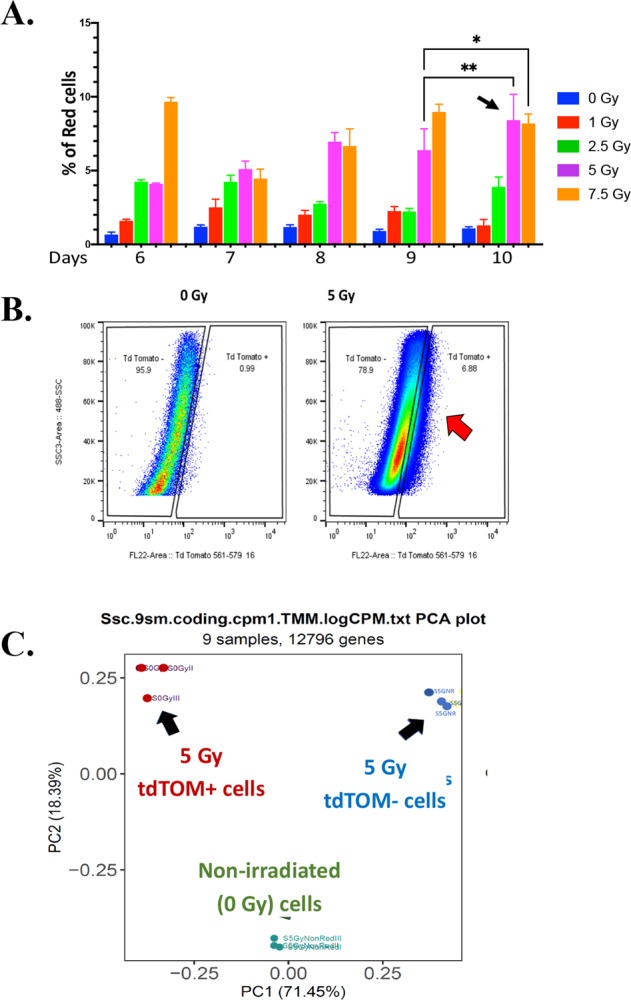
Optimization of in vitro radiation induction of senescent tdTOMp16+ cells. **A** After 10 days, 5-Gy irradiated cells, plated at 50% confluency, yield ~9% of red (tdTOM + ) cells (*n* = 4, *P = <0.5; **P = <0.01. The statistical significance was calculated using one-way ANOVA with multiple comparison). **B** Irradiated (5 Gy) tdTOM+ and irradiated (5 Gy) tdTOM− cells were FACS sorted using gating relative to non-irradiated cells. **C** PCA analysis from RNA-seq using three groups of cells (irradiated tdTOM + , irradiated tdTOM− and non-irradiated cells) show distinct separation in their gene expression and similarity of the three replicates (*n* = 3). We sequenced the transcriptomes of triplicate isolates of three groups of cells: irradiated tdTOM+ senescent cells (0.32 × 10^6^ cells each) replicate, irradiated tdTOM- non-senescent cells (2 × 10^6^ cells), and non-irradiated cells (5 × 10^6^ cells).

**Fig. 2:**
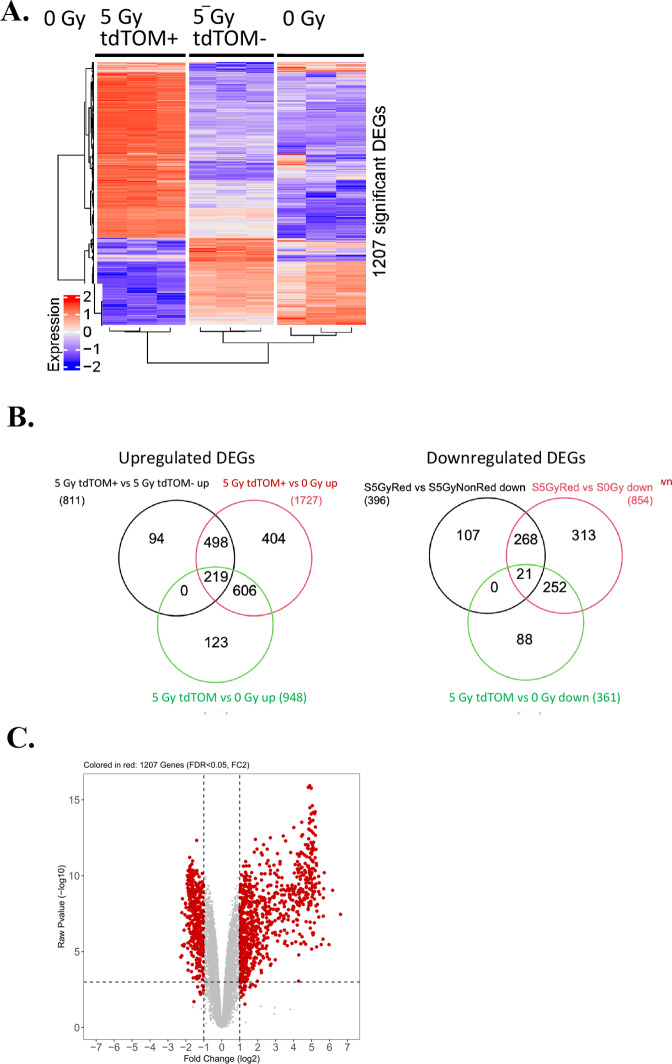
Irradiation-induced tdTOMp16+ senescent cells display a unique transcriptome compared to non-irradiated, or irradiated, but non-senescent cells. **A** The RNA-seq heat map illustrates the expression level of the DEGs between groups. Each row represents a gene and of the nine columns, from left to right, the first three are irradiated senescent (tdTOM + ) samples, the next three columns in the middle are irradiated non-senescent (tdTOM-) samples, and the three columns on the right are non-irradiated samples. The value is the *z-*score of normalized gene expression counts. For any given gene, the red color represents gene expression that is greater than the overall mean, and the blue color represents gene expression that is less than the overall mean. Hierarchical clustering of genes and samples are represented by the dendrograms on the left and across the bottom of the heat map. **B** Venn diagram depicting the shared and unique distribution of the DEGs. On the left are upregulated genes with comparisons between all three groups and on the right are comparisons with downregulated genes. Genes comparing irradiated senescent & irradiated non-senescent samples group are in black circle, irradiated senescent & non-irradiated samples are in red circle, and irradiated non-senescent & non-irradiated samples are in the green circle. **C** Volcano plots show the log2 (fold change) and −log10 (adjusted *P* value) of the genes comparing irradiated senescent and irradiated non-senescent cells from the RNA-seq dataset. The statistically significant DEGs are represented in red. The statistical criteria for a gene to be considered differentially expressed was a fold change ≥ 2 and an adjusted *P* value ≤ 0.05.

We irradiated a mesenchymal stem cell (MSC) line derived from tdTOMp16+ mouse long-term bone marrow cultures [8] to increasing doses at either 50% or 100% cell density. After 10 days, 7–10% of cells at 50% cell density during irradiation, were tdTOM + (red) with both 5 Gy and 7.5 Gy groups (Fig. 1A). Since 7.5 Gy increased cell death, 5 Gy was used for sorting experiments. By FACS, we isolated 7–10% tdTOM+ cells (Fig. 1B). We performed RNA-seq analysis using three independent samples for every three groups: irradiated senescent (tdTOM + ) cells, irradiated non-senescent (tdTOM-) cells, and non-irradiated cells. Large-scale sorting was necessary since 9% of the total irradiated cells were senescent (tdTOM + ) and 90% were non-senescent (tdTOM−). Principal component analysis (PCA) was performed on all genes (12796) present in the RNA-seq dataset and showed the variance of gene expression within and between sample groups. The PCA distribution showed distinct separation of the three groups and all three replicates were clustered together (Fig. 1C). A total of 1207 differentially expressed genes (DEGs) were statistically significant (log2 fold change ≥ 2 and an adjusted *P* value ≤ 0.05) between the irradiated tdTOM+ senescent, irradiated, tdTOM− non-senescent, and non-irradiated cells shown in Fig. 2.

### Radiation-induced tdTOMp16 + senescent cells are non-dividing and are morphologically and biochemically distinct

Since p16 expression is transiently upregulated by DNA damage [20], and one p16 allele is uninterrupted in tdTOMp16+ cells [18], we determined whether the p16 allele and other biomarkers of senescence were induced by irradiation including SA-β-Gal and p21. We observed a similar percentage of tdTOM+ cells were also SA-β-Gal-positive. Furthermore, p16 and p21 were significantly upregulated in tdTOM+ senescent cells compared to irradiated tdTOM− non-senescent cells (Supplemental Fig. 1A, B).

Senescent cells as single cells in 96-well plates were next followed over 4 weeks for cell division. None of the 960 senescent (red) tdTOM+ cells divided after 2 weeks and 137 remained intact. In contrast, 50 of 800 irradiated, non-senescent (non-red) cells divided. Non-irradiated control cells (0 Gy) revealed cell division by 104 of 560 single cells (Supplemental Fig. 2A, B). We observed senescent tdTOM+ cells were larger and circular after 2 weeks compared to tdTOM- cells similar to previously published data [21]. We tracked the motility of 5-Gy irradiated TOMp16+ cells for 2 weeks and observed real-time senescence, including the appearance of td-tomato red fluorescence in cells that also became large and circular (Supplemental Figs. 2C, D and 3A). Quantification of red cells showed a steady increase of tdTOM+ cells after radiation that becomes stable at day 9 (Supplemental Fig. 3B). The movie shows irradiated (5 Gy) tdTOM+ cells turning red over a period of 9 days (Supplemental Fig. 3C). Thus, tdTOM+ red cells showed no cell division and changed shape, while tdTOM− cells that were also irradiated, but non-senescent did divide.

### Distinct gene expression profiles of radiation-induced senescent cells by RNA-seq

To determine if tdTOM+ senescent cells showed a unique pattern of up- or downregulating genes, we analyzed the RNA-seq data as described in Fig. 1C. Cells were sorted into tubes containing tissue culture medium, centrifuged, immediately lysed in Trizol reagent to preserve RNA quality (Supplemental Fig. 4). The relative expression of differentially expressed genes (DEGs) in tdTOM+ senescent cells was clearly distinct from that of the other two groups (Fig. 2A). Furthermore, the irradiated, but non-senescent cells were different from non-irradiated cells (Fig. 2A, B).

The distribution of the differentially expressed genes (DEGs) between the three cell groups is represented in the Venn diagram (Fig. 2B). Of 1944 upregulated DEGs in senescent cells, there were 1811 upregulated gene transcripts in irradiated tdTOM+ vs. irradiated tdTOM- groups. There were 1727 transcripts detected in both irradiated tdTOM+ and non-irradiated groups. There were 948 upregulated transcripts detected in both irradiated non-senescent and non-irradiated groups (Fig. 2B). The volcano plots show the log2 (fold change) versus the −log10 (adjusted *P* value) for all of the genes detected in the RNA-seq analysis (i.e., for both DEGs and non-DEGs; Fig. 2C). The gray color represents genes with no significantly different expression in senescent in tdTOM+ cells, while the red color indicated overexpression or under expression. The data establish, tdTOM+ senescent cells differ from both irradiated non-senescent and control unirradiated cell groups with respect to gene expression patterns.

### Gene transcripts associated with fibrosis are upregulated in radiation-induced senescent cells

**Fig. 3:**
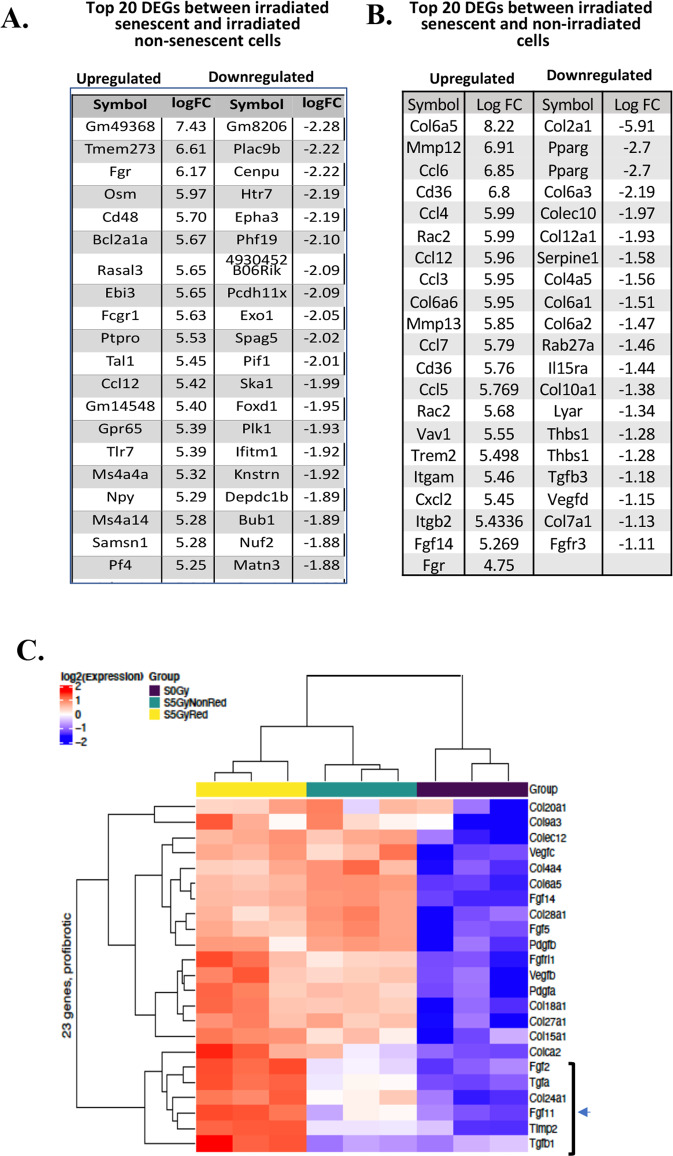
Prominent transcripts in senescent tdTOMp16 + cells include those, which are associated with fibrosis. **A** The top 20 up- and downregulated genes in irradiated senescent & irradiated non-senescent samples. **B** Top 20 up- and downregulated genes in irradiated senescent relative to non-irradiated non-senescent samples. **C** The heat map illustrates the expression level of the differentially expressed profibrotic genes between the three groups. Each row represents a gene and of the nine columns, from left to right, the first three (yellow bar) are irradiated senescent (tdTOM + ) samples, the next three columns (green bar) in the middle are irradiated non-senescent (tdTOM-) samples, and the three columns on the right (black bar) are non-irradiated samples. The value is the *z*-score of normalized gene expression counts. For any given gene, the red color represents gene expression that is greater than the overall mean, and the blue color represents gene expression that is less than the overall mean. Hierarchical clustering of genes and samples is represented by the dendrograms on the left and across the bottom of the heat map.

We list the top 20 overall up- and downregulated differentially expressed genes (DEGs) in senescent cells (Fig. 3A) compared to the irradiated non-senescent tdTOM- cells and control nonirradiated cells (Fig. 3B). The nonreceptor Src-kinase, Fgr was prominently upregulated (6.17-fold) in senescent cells. Other DEGs in senescent cells included Oncostatin M (Osm), Bcl2A1a and Ccl12 (5.97-fold, 5.67-fold, and 5.42-fold, respectively). Osm is involved in p16- and p53-independent Stat3-mediated activation of senescence [22], Bcl2A1a is a member of Bcl2 family which inhibits apoptosis, suggested as necessary for senescent cells to survive [23]. The chemokine Ccl12 is known to recruit fibrocytes in the pathophysiology of lung fibrosis [24]. Among the top downregulated genes in senescent cells were the centromere protein U (Cenpu), EphA3, and Foxd1 (−2.22, −2.19, and −1.95-fold, respectively). Cenpu is known to be underexpressed in senescent cells [25], EphA3 has been shown to inhibit senescence [26]. Since Foxd1 upregulation by YAP decreases senescence [27], downregulation in our senescence cells was expected. Figure 3B shows the top 20 up and downregulated genes in senescent cells relative to non-irradiated cells. Except for Ccl12, these genes were different from the DEGs found when comparing senescent cells with irradiated non-senescent cells. Fgr was not among those top 20 but was upregulated 4.75-fold. Further investigation of the decline in Fgr induction (−1.42-fold) when comparing irradiated senescent vs non-irradiated cells (4.75-fold) as opposed to irradiated senescent vs irradiated non-senescent cells (6.17-fold) revealed that the decline is slight and likely owing to the baseline tdTOMp16 expression in non-irradiated cells (Supplemental Fig. 5).

We next compared 23 known profibrotic genes between tdTOM+ senescent cells, tdTOM- irradiated, but non-senescent cells, and control non-irradiated cells (Fig. 3C). The heat map shows that profibrotic genes were significantly upregulated in tdTOM+ cells compared to irradiated non-senescent and control non-irradiated cells. Some profibrotic genes were detectably induced in irradiated non-senescent cells relative to control non-irradiated cells. The major targets including Tgfβ1, Tgf-α, Fgf2, Timp2 were induced in tdTOM+ senescent cells (Fig. 3C, arrow), but not in the irradiated but non-senescent cells.

### Senescent cells induce biomarkers of fibrosis in target cells in transwell culture

**Fig. 4:**
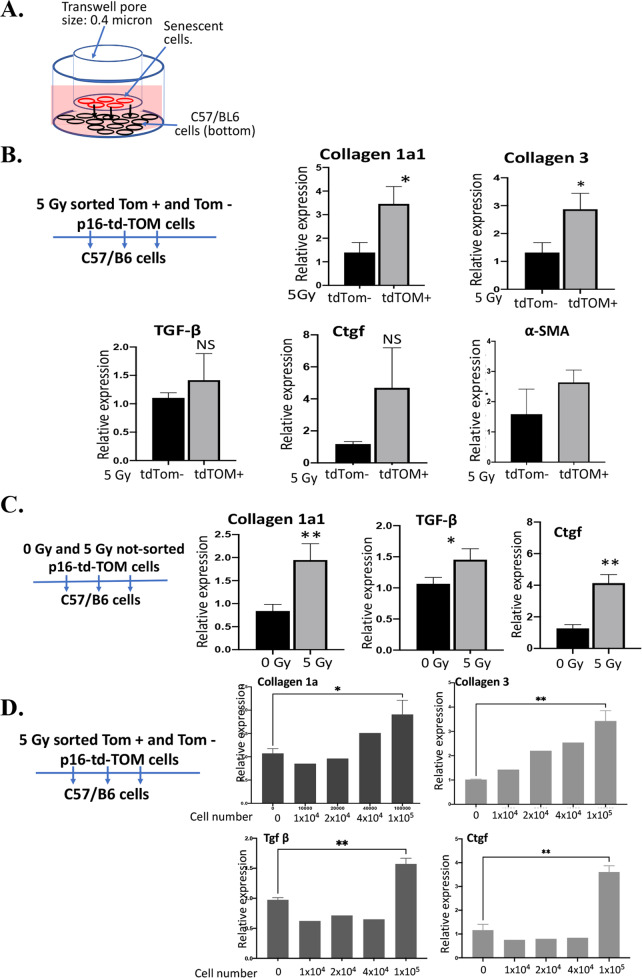
Senescent tdTOMp16 + cells induce biomarkers of fibrosis in target cells. Scheme of non-contact transwell coculture system. The top well and the bottom well were: **A** separated by 0.4-micron filter. **B** Irradiated tdTOMp16+ senescent (tdTOM + ) and non-senescent (tdTOM−) cells were cultured on top well and the target C57BL/6 stromal cells were cultured on the bottom well. After 10 days, target cells were harvested for RNA and RT-qPCR was performed for Collagen1a1, Collagen 3, and TGF-β, CTGF, and α-smooth muscle actin (α-SMA) genes. **C** Irradiated (5 Gy) and non-irradiated (0 Gy) tdTOMp16+ cells were cultured on top well and the target C57BL/6 stromal cells were cultured on the bottom well. After 10 days, target cells were harvested for RNA, and RT-qPCR for Collagen1a1, TGF-β, and CTGF genes were performed. **D** Increasing number of senescent tdTOMp16+ cells (0, 1 × 10^4^, 2 × 10^4^, 4 × 10^4^, and 1 × 10^5^ cells) were plated on top well and the target C57BL/6 stromal cells were cultured on the bottom well. After 10 days, target cells were harvested for RNA and RT-qPCR for Collagen1a1, Collagen 3, TGF-β, and CTGF genes was performed (*n* = 3, *P = <0.05; **P = 0.01, NS = non-significant). *P* values were calculated by *t* test.

To determine whether humoral factors were released by senescent cells, induced biomarkers of fibrosis, we tested effects on target cells in a transwell coculture system where target cells were separated from senescent cells by a 0.4-micron membrane (Fig. 4A). We plated in the top chamber of each culture irradiated, sorted tdTOM+ cells compared to irradiated, sorted tdTOM− cells with irradiated non-sorted cells. We placed in the bottom layer of each culture either MSC target cells (Fig. 4B, C) or primary mouse tail fibroblasts (Supplemental Fig. 6). We quantitated gene induction levels in target cells from the bottom wells by using RT-qPCR. We observed upregulation of TGF-β, CTGF, Collagen 1, Collagen 3, and α-smooth muscle actin in target cells by irradiated tdTOM+ cells compared to non-irradiated cells (Fig. 4 and Supplemental Fig. 6).

To determine whether irradiation-induced induction of profibrotic genes in target cells by senescent cells was inhibited by the presence of irradiated non-senescent cells, we tested unsorted irradiated cells in the transwell cultures. These populations also induced biomarkers of fibrosis in target cells including TGF-β, CTGF, Collagen 1, Collagen 3, α-smooth muscle actin (Fig. 4C). These results establish that 9% of senescent cells were active in inducing biomarkers of fibrosis even in the presence of 90% non-senescent cells.

We next quantitated the effect of cell number on the induction of biomarkers of fibrosis. We plated sorted senescent tdTOM+ cells in several numbers to determine the minimal number required to stimulate fibrotic gene expression in target cells. A minimum number of 10^4^ senescent cells were required to induce a detectable induction of Collagen 1α, and Collagen 3 in target cells in the transwell cultures. This induction was elevated by increasing numbers of tdTOM+ cells in a direct dose-response relationship. A minimal number of 10^5^ tdTOM+ cells was required to induce TGF-β and CTGF (Fig. 4D). The data indicate that purified irradiated tdTOM+ senescent cells induce biomarkers of fibrosis in target cells in a non-contact transwell system which is in line with our finding that TGF-β and collagen were also induced in the lungs of RIPF lungs [6, 28].

### Tyrosine kinase Fgr in senescent cells induces biomarkers of fibrosis

We next confirmed that upregulation of Fgr in senescent cells, as determined by RNA-seq correlated with overexpression of Fgr by RT-qPCR (Supplemental Fig. 7A). We also measured levels of SASP genes including Cc13, Cc112, and Ccl4 that were induced by radiation in senescent cells (Supplemental Fig. 7B).

**Fig. 5:**
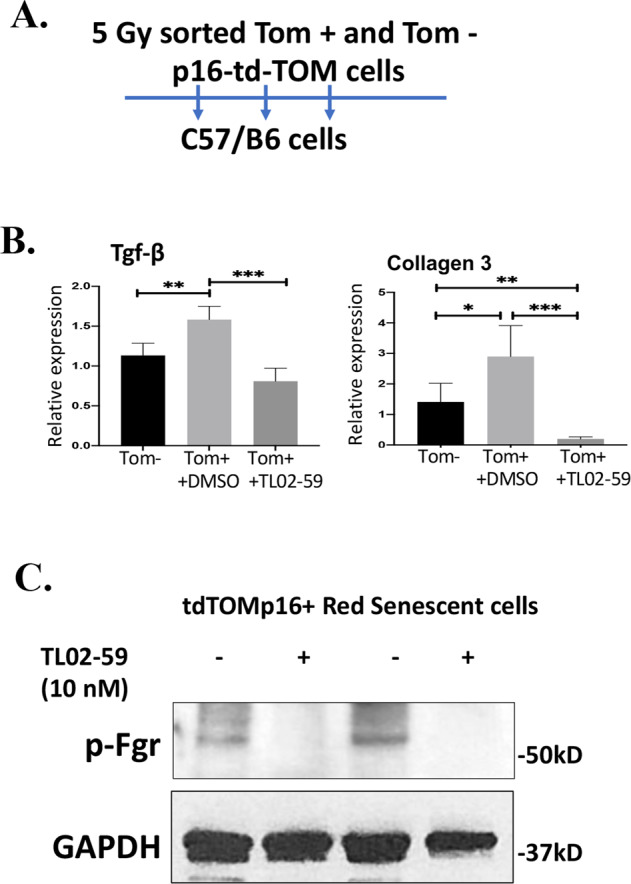
In transwell culture, inhibition of tyrosine kinase Fgr by TL02-59 abrogates the induction of profibrotic genes in target cells. **A** Irradiated tdTOMp16+ non-senescent (tdTOM−) and senescent (tdTOM + ) cells were cultured on the top well of the transwell, and the target C57BL/6 stromal cells were cultured on the bottom well. Cells were treated with either vehicle or 10 nM Fgr inhibitor TL02-59 and after 10 days, target cells were harvested for RNA, and RT-qPCR was performed for TGF-β and Collagen 3 genes. **B** Inhibition of Fgr phosphorylation by TL02-59 was confirmed by western blot from the vehicle and TL02-59-treated cell lysates of irradiated senescent (tdTOM + ) cells (*n* = 3, *P = <0.05; **P = 0.01). **C** Phosphorylation of Fgr was inhibited by TL02-59. NS non-significant), *P* values were calculated by *t* test.

The role of Fgr in fibrosis induction was next investigated by two methods of inhibition: we first applied pharmacologic inhibition of Fgr by adding the Fgr-specific small-molecule inhibitor TL02-59 (Fig. 5A, B). Parallel KINOMEscan analysis, in vitro kinase assays, target kinase gene expression profiling, and efficacy studies identified the Src-family kinase Fgr as the primary target for TL02-59 [19]. Using the transwell non-contact coculture system, we measured the effect of a specific inhibitor of Fgr TL02-59 (10 nM) on the induction of biomarkers of fibrosis in target cells by tdTOM+ senescent cells for 10 days. We treated the cells with 10 nM TL02-59 following a published protocol [19]. We determined by RT-qPCR that TL02-59 significantly inhibited induction of collagen 3 and TGF-beta (Fig. 5B). We confirmed that TL02-59 inactivated Fgr by phosphorylation in senescent cells using western blotting and a phosphor-Fgr antibody (Fig. 5C).

### shRNA silencing of Fgr does not block irradiation-induced senescence but suppresses induction of fibrosis biomarkers in target cells

**Fig. 6:**
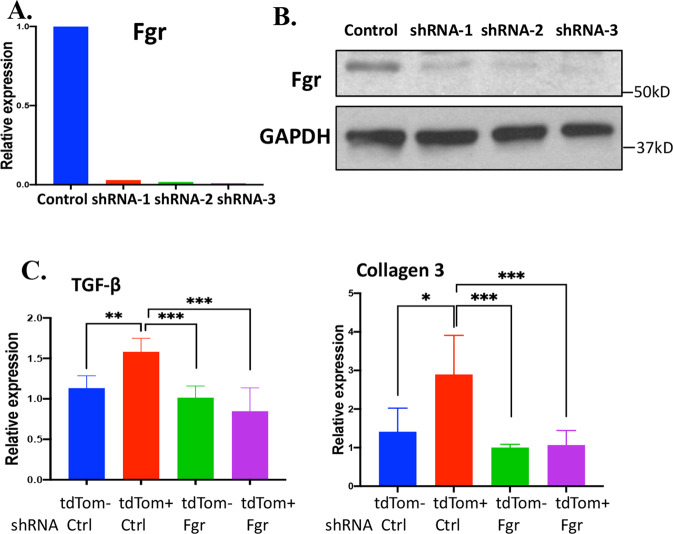
shRNA inhibition of Fgr in senescent (tdTOM + ) cells abrogates the induction of profibrotic genes. **A** Validation of the efficacy of 3 shRNAs by RT-qPCR targeting the coding region of Fgr after 72 h of transient transfection in mouse bone marrow stromal cells. **B** Validation of the efficacy of three shRNAs by western blotting after 72 h of by transient transfection in mouse bone marrow stromal cells. **C** Stable control or Fgr shRNA knockdown tdTOMp16+ cells were irradiated and sorted non-senescent (tdTOM−) and senescent (tdTOM + ) cells were cultured on top well of the transwell, and the target C57BL/6 stromal cells were cultured on the bottom well and after 10 days, target C57BL/6 cells were harvested for RNA and RT-qPCR was performed for TGF-β and Collagen 3 genes. (*n* = 3, *P = <0.05; **P = 0.01, NS non-significant), *P* values were calculated by *t* test.

The Fgr inhibitor, TL02-59, might affect both the senescent cells on the top of the transwell well and the target cells at the bottom well in the transwell. Next, we constructed a stable Fgr shRNA expressing subline of the MSC cell line. We tested candidate-Fgr shRNAs by transient transfection into tdTOMp16 bone marrow stromal cells and measured efficacy after 72-h by RT-qPCR and western blots (Fig. 6A, B). We selected shRNAs that produced over 80% Fgr silencing and derived stable cell lines by puromycin resistance. We then tested whether irradiated Fgr-silenced cells became senescent. At 10 days after 5 Gy irradiation, there was no detectable reduction in the 7–10% cell senescence by Fgr shRNA knockdown (silenced) in tdTOMp16 cells compared to control tdTOMp16 cells. Furthermore, RT-qPCR showed, p16 and p21 levels were increased in irradiation-induced shRNA knockdown senescent cells. However, Fgr-silenced tdTOMp16 red senescent cells did not induce TGF-β or collagen 3 (biomarkers of fibrosis) in target cells after 10 days in transwell cultures (Fig. 6C).

### Lung irradiation induces Fgr in senescent cells prior to detection of RIPF

**Fig. 7:**
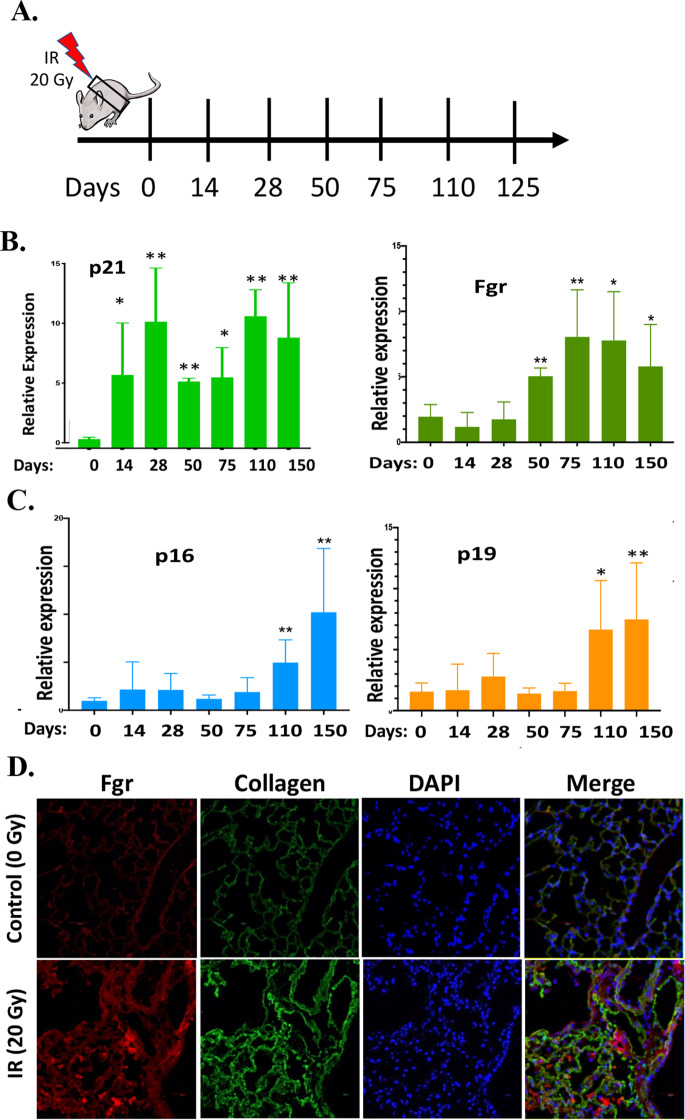
Induction of Fgr and biomarkers of senescence in irradiated mouse lungs. **A** 951 Scheme showing the thoracic irradiation (20 Gy) and the days the lungs of the C57BL/6 mice 952 were harvested for total RNA. **B** Expression of p21 and Fgr in the mouse lungs were evaluated 953 by RT-qPCR at different days. **C** Expression of p16 and p19 in the mouse lungs was evaluated by RT-qPCR at different days. **D** 20 Gy irradiated mouse lungs tissue was immunostained using Fgr and Collagen antibodies (*n* = 3−5 mice, *P = <0.05; **P = 0.01), *P* values were calculated by ANOVA test).

Prior studies showed senescent cells were detected by β-gal and p16 staining of lungs at day 75 after 20 Gy thoracic irradiation and before detection of fibrosis at day 150 [6]. We quantitated levels of Fgr compared with p21, p16, and p19 in the lungs of 20 Gy thoracic-irradiated mice over 150 days (Fig. 7A). Fgr was upregulated in 20 Gy irradiated lungs at day 50 (Fig. 7B), while p16 and p19 were increased at 110 days (Fig. 7C), before detectable fibrosis at day 150. Both Fgr and collagen were significantly induced in the fibrotic lungs at day 150 after irradiation compared to no-irradiation control lungs as evidenced by immunohistochemistry (Fig. 7D).

### Single-cell RNA sequencing (scRNAseq) identifies Fgr induction in monocytes, macrophages, and neutrophils in thoracic-irradiated lungs

**Fig. 8:**
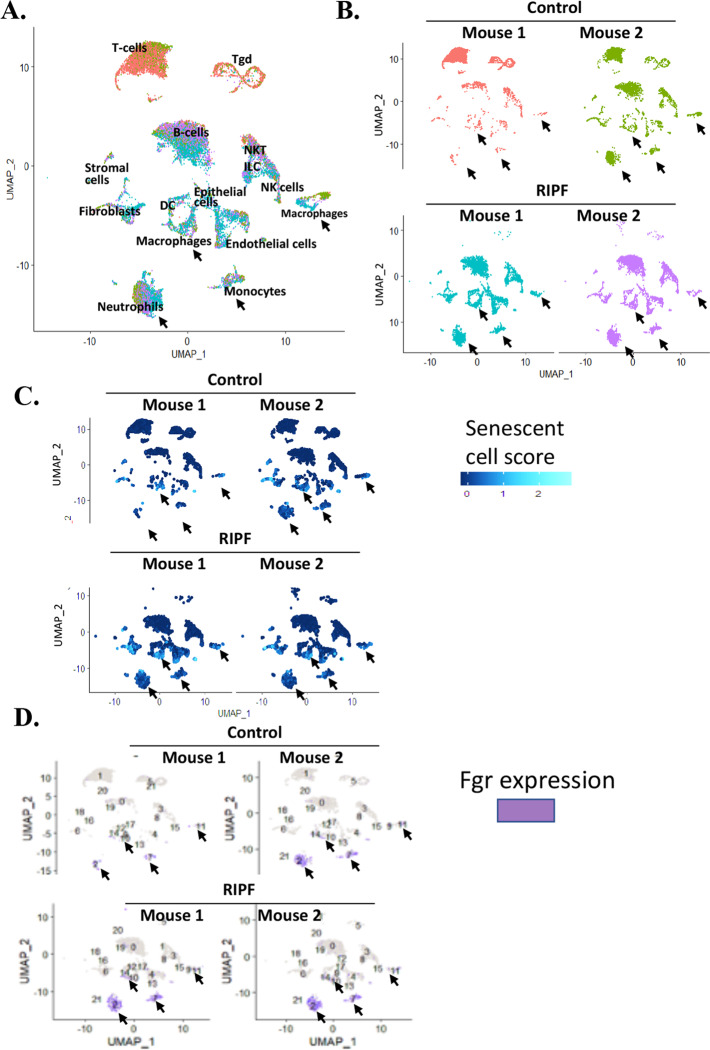
Single-cell RNA-Seq analysis of mice with radiation-induced pulmonary fibrosis. **A** Single-cell RNA-Seq was performed on single-cell suspensions generated from two control and two 150 days post-irradiated (20 Gy) irradiated mouse lungs with fibrosis. All samples were analyzed using canonical correlation analysis within the Seurat R package. Cells were clustered using a graph-based shared nearest-neighbor clustering approach and visualized using a U-map plot. Canonical cell markers were used to label clusters by cell identity as represented in the plot. Cell types were classified as indicated. **B** Cellular populations represented in all four samples are shown in UMAP plot identified. Each sample was color-coded to show the differences in cell types represented by control and irradiated lungs. The distribution of senescent cells is shown for both control and irradiated lungs cell types in the UMAP plot. We used the expression of canonical 23 senescence-associated gene expressions to generate a senescence score for any individual cell and applied it to all cell types in our dataset. The lighter the blue, the more the expression of the genes. **C** Distribution of Fgr expressing cells is shown for both control and irradiated lung cell types in the UMAP plot. **D** Purple color indicates the expression of Fgr.

To identify the cell phenotypes that overexpressed Fgr in RIPF, we performed scRNAseq on cells from irradiated mouse lungs. Thoracic-irradiated mice were sacrificed at 150 days, lungs removed, and single cells isolated. A total of 19879 cells were used for integrated single-cell RNA-seq analysis. We assigned cell types to each cluster based on the expression of established markers from the LungMAP and IMMGen databases (Supplemental Fig. 8). There were identifiable epithelial cells, endothelial cells, macrophages, monocytes, neutrophils, dendritic cells; T cells, natural killer cells, B cells, stromal cells, and fibroblasts (Fig. 8A, B). The cell phenotypes in two control mice and two 150-day irradiated mice are shown. Using a panel of 23 senescence-associated genes (Supplemental Fig. 9), senescence markers were detected in macrophages and neutrophils (Fig. 8C). ScRNAseq analysis revealed Fgr was present in monocytes, neutrophils in control lungs. At 150 days after 20 Gy irradiation, Fgr was significantly upregulated in neutrophils, a cluster of senescent cell macrophages, and in dendritic cells (Fig. 8D and Supplemental Fig. 10).

## Discussion

In these studies, we purified radiation-induced senescent cells. A cell line was derived from tdTOMp16+ bone marrow in which the red tomato (tdTOM) fluorochrome is attached to exon1 of one allele of the p16 gene while the other allele is uninterrupted [18]. Cells irradiated to 5 Gy, showed 7–10% senescent cells at day 10. Irradiation induces DNA-damage-driven senescence including upregulation of CDK1 p16 [29].

RIS (tdTOM + ) cells were compared with non-senescent (tdTOM−) cells in the same cultures. Senescent cells were clearly different from irradiated non-senescent cells and control non-irradiated cells by RNA-seq. PCA and the heat maps indicated that levels of 23 profibrotic genes were increased in senescent cells including TGF-beta, Tgf-alpha, TIMP2, Fgf2, Fgf11, and Col24a1. The tyrosine kinase Fgr was Log2 6.17-fold upregulated in RIS cells, which induced fibrosis biomarkers in target cells, separated in transwell cultures. Inhibition of Fgr by shRNA knockdown or adding an inhibitor of Fgr to the cultures blocked the induction of fibrotic biomarkers.

Fgr kinase has been implicated in proinflammatory adipose tissue macrophage activation, diet-induced obesity, insulin resistance, and liver steatosis [30]. Fgr is known to be induced in some patients with acute myeloid leukemia, and inhibition of Fgr by TL02-59 clears transplanted leukemic cells in mice [19]. Tyrosine kinase activity is known to disturb physiological homeostasis and lead to cancer, vascular disease, and fibrosis. In fibrosis, receptor tyrosine kinases like PDGF receptor (PDGFR), VEGF receptor (VEGFR), EGF receptor (EGFR), and JAK kinases and nonreceptor tyrosine kinases such as e.g., c-Abl, c-Kit, and Src kinases have been identified as determinants of disease progression and potential targets for anti-fibrotic therapies [31–34]. Specifically, Fgr deficiency has been linked to the reduced secretion of chemokines in the lungs in response to lipopolysaccharides [35, 36].

Senescent cells produce reactive oxygen species (ROS) [37], which activate the tyrosine kinase Fgr. We identified Fgr upregulation in irradiated mouse lungs, and the increase was detected at day 50 significantly before detection of fibrosis at day 150. By scRNAseq from mouse lungs, we showed that Fgr is predominantly expressed in macrophages, monocytes, and in neutrophils. In RIPF, we observed macrophage senescence and a distinct cluster of macrophages appears where Fgr is upregulated. At present, we do not know if Fgr is unique from other tyrosine kinases with respect to the induction of RIPF [30]. Ablation of Fgr is known to impair proinflammatory macrophage polarization [37]. In prior studies, using scRNAseq, four separate macrophage clusters were present in IPF patient’s lungs. Two macrophage clusters were represented equally in normal and fibrotic lungs, while two other clusters were found only in the lungs of IPF patients [38]. Senescent fibroblasts and type II airway epithelial cells and macrophages have been reported to accumulate in the lungs during the induction of RIPF [27, 39, 40]. Fgr is known to be upregulated in the lungs in idiopathic pulmonary fibrosis (IPF), and is primarily expressed in macrophages, monocytes, and dendritic cells [41]. Our data are consistent with prior publications emphasizing the role of monocyte/macrophages in lung fibrosis and extend the data to RIPF. Future studies are needed to understand whether Fgr is involved universally in all kinds of senescence-mediated fibrosis.

Senescence may impair healing via the CXCR2 receptor, which, impedes repair [42, 43]. A major ligand of CXCR2 is Cxcl2, which is a SASP component. In our RNA-seq analysis, Cxcl2 was induced in senescent cells. Since SASP recruits macrophages to sites of lung injury, such cells may impair tissue healing [39, 44]. SASPs can induce paracrine senescence in neighboring cells; thereby, forming the generation of reactive oxygen species (ROS), and the DNA-damage response [45]. Paracrine senescence may exacerbate the biliary injury and impair regeneration in the liver [46]. Targeted reduction of SASP, through TGF-β inhibition helps restore hepatocyte proliferation, decreases fibrosis, and improves, overall, liver function [47].

Removal of senescent cells to prevent late radiation fibrosis may seem logical [12, 28, 48–50]; however, senolytic drugs may not target all senescent cells [32–34]. Senolytic drugs Dasatinib plus Quercetin in IPF patients showed no changes in pulmonary function and had unclear effects on reducing SASP [42]. Dasatinib promotes apoptosis [51] by inhibiting Src kinases [52]. Quercetin [53], a naturally occurring flavonoid acts in part by inhibiting BCL‐2 family members. While Dasatinib plus Quercetin is effective in preventing aging in mouse models, neither alone [31, 53] are senolytic because of the complexity of their targets.

Alternatively, retaining senescent cells may provide valuable functions including restraining tumor growth [54], stimulating wound healing [55], and promoting tissue repair [56].

If removal of specific components of SASP can be achieved, it is possible that retaining senescent cells may help restore and repair fibrotic lungs from radiation damage.

Other approaches to treat irradiation pulmonary fibrosis include pirfenidone and nintedanib, which slow the progression of fibrosis in the lung [57, 58]. Pirfenidone works through the inhibition of TGF-β [59]. Nintedanib is a tyrosine kinase inhibitor for fibroblast growth factor receptor (FGFR)-1 and vascular endothelial growth factor receptor (VEGFR) [60]. The efficacy of these agents is poor [61, 62] and their effects on senescent cells have not been studied in detail.

These studies identified a novel mechanism of RIPF mediated by increases in profibrotic Fgr in senescent cells. We demonstrated that a small-molecule inhibitor of tyrosine kinase Fgr blocks the radiation fibrosis pathways. Studies evaluating the reduction of RIPF by TL02-59 treatment of thoracic-irradiated mice are in progress. Senescent cells and their induction of tyrosine kinase Fgr may play roles in lung fibrosis originating from causes other than RIPF. The therapeutic potential of inhibitors of Fgr in RIPF and other causes of lung fibrosis including after COVID-19 infection merit further study.

## Materials and methods

### Mice

The mouse strain tdTOMp16+ was obtained from Dr. B. O. Diekman, University of North Carolina at Chapel Hill [18], and C57BL/6 mice were purchased from The Jackson Laboratory. Thoracic irradiation was carried out according to published methods [8]. For the mouse thoracic irradiation experiment, a total of 34 C57BL/6 mice were randomly divided into control and experimental groups and sacrificed at indicated time points. All animal protocols used were approved by the University of Pittsburgh’s IACUC and veterinary care was being provided.

### Materials

Non-target control (sh-C018) and 3 shRNAs targeting mouse Fgr gene were purchased from (Sigma-Aldrich, St. Louis, MO) (shRNA #1, Clone ID: NM_010208.4-328s21c1, Sequence: CCGGACGG CTGAAGAACGCTATTTCCTCGAGGAAATAGCGTTCTTCAGCCGTTTTTTG; shRNA #2: Clone ID: NM_010208.2-758s1c1, Sequence: CCGGGCGATCACAT AAAGCATTATACTCGAGT ATAATGCTTTATGTGATCGCTTTTT and ShRNA #3, Clone ID: NM_010208.2-853s1c1 Sequence: CCGGCGGCACTACATGGAAGTGAATCTCGAGATTCACTTCCATGTAGTG CCGTTTTT), Rabbit anti-Fgr (# sc-74542) antibody was purchased from Santa Cruz Biotechnology, Inc and anti-phospho-Fgr antibody was purchased from the Invitrogen (Catalog # PA5-64583). Antibody to GAPDH was purchased from Sigma-Aldrich (St. Louis, MO). Antibody to collagen I was purchased from Abcam (Waltham, MA) (ab21286). TL02-59 was purchased from MedChemExpress as powder form (Cat. No.: HY-112852).

### Long-term bone marrow cultures

Long-term bone marrow cultures were established according to previously published methods [63–65]. The contents of a femur and tibia of tdTOMp16 + (heterozygous) mice [18] were flushed into a 40 cm. square plastic flask in Dulbecco’s modified Eagle’s medium containing 25% fetal calf serum and 10^−5^ M hydrocortisone sodium hemisuccinate and antibiotics. Cultures were medium-changed weekly with the removal of nonadherent cells and replacement with an equal volume of fresh medium. Cultures were maintained in a high humidity incubator with 7% C0_2_ and medium-changed weekly. Stable shRNA knockdown cells were made by transfecting shRNA plasmids, no-target-control plasmids transfected by Lipofectamine 3000 and were selected against puromycin (4 μg/ ml) as mixed clones.

### Bone marrow stromal cell lines

Adherent cell monolayers from bone marrow cultures were maintained in Dulbecco’s modified Eagle’s medium supplemented with 20% fetal bovine serum and passaged weekly. Cell lines were derived from a 4-week culture harvest of the adherent layer and were maintained at 37 °C [65].

### Irradiation dose and time experiment

tdTOMp16+ bone marrow stromal cells were cultured in a 24-well plate at 50 and 100% confluency and the cells were exposed to 0, 1, 2.5, 5, and 7.5 Gy (n = 4). For 10 days the tdTOM+ cells were imaged using Olympus BAX Motorized Fluorescence Microscope. The images were counted using ImageJ software.

### In vitro cell sorting

Serial-passaged tdTPMp16+ bone marrow stromal cell line, either mock irradiated or 10 days after 5 Gy irradiation were sorted by FACS into tdTom− and tdTom+ populations using MoFlo XDP (Beckman Coulter) or FACSAria III (Becton Dickinson). For FACS gating, tdTOMp16+ Sorted cells were then used to (1) plate as single cells in each of the wells of 96-well plates [65] RNA-seq experiment, or [65] the functional studies using transwell coculture method section.

Cell division monitoring: non-irradiated and 5 Gy Irradiated cells for both tdTOM+ and tdTOM- subpopulations were sorted. Cells were plated 10-day post irradiation as a single cell in each well of the 96-well plate (*n* = 8–10). The cells were monitored for cell division for 4 weeks.

### Stable shRNA knockdown of Fgr

tdTOMp16 cells were cultured at 70% confluency and 24 h after the cells were transfected with Lipofectamine 3000 reagent using non-target control and Fgr shRNAs. Transfected cell lines were sub-cultured after 24 h of transfection, and puromycin was added at 4 μg/ ml concentration. The culture media of the transfected plates were replaced with fresh media to eliminate dead non-resistant cells. Once the resistant cells reach 80% confluency, cells were sub-cultured and the remaining cells were processed for Fgr knockdown validation by RT-q-PCR and western blotting.

### Transwell coculture experiments

A Transwell system (0.4-μm pore size, polyester membrane; Corning, Kennebunk, ME) was used [66]. C57BL/6 mouse bone marrow stromal cells or mouse primary tail fibroblasts were cultured in the bottom surface of a 9-cm^2^ culture dishes. Above the adherent layer in the transwell, tdTOMp16+ bone marrow stromal cells (non-irradiated, irradiated non-sorted, irradiated, and sorted senescent tdTOM + , or irradiated non-senescent tdTOM- cells) were cultured in the upper transwell. For the TL02-59 experiment, the drug (10 nM) was added to the media at the time the cells were plated on the transwell. For the stable Fgr shRNA cells, non-target control and Fgr shRNA knockdown cell lines were also irradiated and sorted following the same protocol. Briefly, tdTOMp16+ cells were irradiated and after 10 days of radiation, the cells were FACS sorted for tdTOM+ and tdTOM- cells and 3 × 10^5^ cells added on the top wells in each case. Coculture was maintained for another 10 days. The target cells from the bottom well were lysed, and total RNA was isolated using TRIzol (Sigma) reagent following the manufacturer’s protocol. cDNA was synthesized, and expression of profibrotic genes (TGF-β1, CTGF, collagens (Col1, Col3), and α-SMA were analyzed using gene-specific TaqMan primers employing quantitative real-time polymerase chain reaction (PCR).

### RNA isolation and cDNA synthesis

Total RNA was isolated from respective cell lines (tdTOMp16 + bone marrow stromal cell line, C57BL/6 bone marrow stromal cell line, and mouse primary tail fibroblasts) according to the protocol supplied with TRIZOLl Reagent (Invitrogen, Life Technologies, Thermo Fisher Scientific, Waltham, MA). The concentrations of the RNA samples were determined using a spectrophotometer and cDNAs were made from RNA (2 μg) using high-capacity RNA-to-cDNA™ Kit (Thermo Fisher Scientific) following the manufacturer’s instructions.

### Real-time PCR

Quantitative reverse transcription-PCR (qRT-PCR) was performed using Biorad CFX-connect Real-Time System instrument and commercially available target probes and Master mix (all from Applied Biosystems). Detection of mouse Fgr, p16 (CDKN2A), p21 (CDKN1A), p19, Collagens (1and 3), TGF-beta, α-smooth muscle actin (Acta 2), CTGF and GAPDH were achieved using specific Taqman Gene Expression Assays (Mm00438951_m1, Mm00494449_m1, Mm04205640_g11, Mm01191861_m1, Mm01192933_g1Mm01257348_m1, Mm00600638_m1, Mm00725412_s1, Mm00802305_g1, Mm99999915_g1, respectively). Real-time reactions were run using the following cycling parameters: 95 °C for 12 min, followed by 40 cycles of 95 °C for 15 s and 60 °C for 1 min. Differential gene expression was calculated by the ΔΔCT calculation [38].

### RNA library construction and next-generation sequencing

RNA libraries were prepared from three groups of tdTOMp16+ cells consisting of: (1) non-irradiated cells (*n* = 3); (2) irradiated senescent tdTOM + (red) cells (*n* = 3); and, (3) irradiated non-senescent tdTOM- cells (*n* = 3). Compiled RNA-seq data was used in the analysis of Figs. 1 and 2. To minimize the effect of any RNA degradation present in the samples, libraries for RNA-seq were generated from ribosomal RNA depleted total RNA rather than from mRNA isolated by poly-A selection. One microgram of each DNase I-treated RNA sample was used for library construction using the Illumina TruSeq Stranded Total RNA Library Prep Kit with Ribo-Zero Gold per the manufacturer’s protocol. Adaptor-ligated fragments were amplified by PCR for nine cycles. The quality and size of the final library preparations were analyzed on an Agilent TapeStation. Sequencing was performed on the Illumina NextSeq. 500 NGS platform, generating ~40 million paired-end 75 bp reads for each sample. The BioSample accession code for the deposited RNA-seq data to NIH is SAMN22069450.

### Differential gene expression (DEG) detection and gene set enrichment analysis (GSEA) from RNA-seq data

The RNA-seq data were processed following previously established protocols [67]. Raw reads were assessed for quality using FastQC [68] (v0.11.7). Transcript-level abundance was quantified using Kallisto [69] (v0.46.1) in the reverse strand-specific mode with mouse reference assembly (GRCm38) and Gencode gene annotation (v25), and summarized into gene-level using tximport [70] (v1.16.1). Lowly expressed genes (counts per million reads mapped (CPM) < 1) were removed, and data were normalized across all samples using Trimmed Mean of M-values (TMM) method. Significant DEGs were identified using limma voom [71] (v3.46.0) with precision weights and filtered by fold change ≥2.0 or ≤ −2.0 and FDR-adjusted *P* < 0.05. GSEA was performed using R package fGSEA (v1.14.0) with gene sets of interest (chemotaxis, senescence, and profibrotic). Pathway enrichment of DEGs was detected using Ingenuity Pathway Analysis^®^ (IPA) with Ingenuity Knowledge Base (QIAGEN, Inc).

### Generation of single-cell suspensions from whole mouse lung

The lungs of mice were first inflated with 1 ml of sterile PBS and allowed to collapse, and then the lungs were inflated with the enzyme mix containing dispase (50 caseinolytic units/ml), collagenase (2 mg/ml), elastase (1 mg/ml), and DNase (30 ug/ml). The lungs were removed and immediately minced into small pieces (~1 mm^2^). The tissue was transferred into 10 ml enzyme mix for enzymatic digestion for 30 min at 37 °C. Enzyme activity was inhibited by adding 5 ml of phosphate-buffered saline (PBS) supplemented with 10% fetal calf serum (FCS) [72]. Dissociated cells in suspension were passed through a 70-µm strainer and centrifuged at 500 × *g* for 5 min at 4 °C. Red blood cell lysis (Thermo Fisher 00-4333-57) was done for 2 min and stopped with 10% FCS in PBS. After another centrifugation for 5 min at 500 × *g* (4 °C), the cells were counted using a Neubauer chamber and critically assessed for single-cell separation and viability. A total of 250,000 cells were aliquoted in 2.5 ml of PBS supplemented with 0.04% of bovine serum albumin and loaded for DropSeq at a final concentration of 100 cells/µl.

### ScRNAseq

For scRNAseq, we used Chromium Next GEM Single Cell 3’ Reagent Kits v3.1 (10X Genomics, Pleasanton, CA) and followed the manufacturer’s protocol [73]. Briefly, cells are loaded onto a chip, run through the Chromium device, where they were encapsulated into individual oil droplets containing 10X Genomics’ barcoded gel beads forming GEMs (Gel bead in Emulsion). These were then collected, and reverse transcription was performed, producing cDNA that contained the gene transcript with an associated cell barcode and unique molecular identifier. The cDNA was then purified, amplified, and libraries were prepared. Libraries were dual-indexed containing both a 17 and 15 index [74].

The quality of raw reads was assessed using FastQC. The feature-barcode count matrices were generated from FastQ files using the CellRanger pipeline (v6.0.1; 10X Chromium Single Cell 3’ V3 chemistry). CellRanger maps reads against mouse genome GRCm38 using STAR aligner, followed by Unique Molecular Identifier (UMI) counting with sequencing error correction and call barcode calling using the “EmptyDrops” method [75]. Downstream analysis was performed using Seurat (v3.9.9.9024) to import feature-barcode hdf5 files, filter cells to keep those with 200–5000 features (genes), and mitochondria gene content <15%, keep genes present in >3 cells and normalize data by the “LogNormalize” method. A total of 19,879 cells and 25,319 genes were kept for analysis. Uniform Manifold Approximation and Projection [76] (UMAP) was generated using Principal Component Analysis (PCA) dimension reduction with scaled data. Cell clusters were identified using a shared nearest-neighbor (SNN) modularity optimization-based clustering algorithm and visualized on UMAP. Cell type assignment was performed using SingleR [77] (v1.2.4) with the mouse Immunological Genome Project (ImmGen) as the reference database. Annotated cell types were manually inspected using known immune markers. Cell cycle phases were scored using genes in the “cc.genes.updated.2019” object after converting human gene symbols to the mouse by biomaRt (v2.44.4). The BioSample accession codes for the deposited scRNAseq data to NIH are SAMN22071902, SAMN22071903.

### Immunohistochemistry

C57BL/6 received either 0 or 20 Gy irradiation to the thoracic cavity. Thoracic-irradiated mice were sacrificed at day 150 post irradiation. Explanted tissues were frozen in optimal cutting temperature (OCT), sectioned and immunochemistry was performed using antibodies for Fgr (Santa Cruz Biotechnology, Inc # sc-74542) and collagen I (Abcam, ab21286). Five randomly selected images were captured in a blinded fashion from each section using fluorescent confocal microscopy.

### Statistics

For the analysis of the percent of red cells, we used two-way ANOVA, where radiation dose, day, and their interaction are factors, followed by post hoc *t* tests. For the RT-qPCR analysis of gene expression, and for the western blot analysis, we used one-way ANOVA followed by post hoc *t* tests. For the other two group comparisons, we used two-sample *t* tests or Wilcoxon rank-sum tests, where appropriate. *P* values less than 0.05 were regarded as significant. In these exploratory analyses, we did not adjust *P* values for multiple comparisons.

## Supplementary information

Supplemental material

Real time movie of radiation-induced senescent cell formation

## Data Availability

RNA-seq and Single-cell RNA sequencing data that support the findings of this study have been deposited in SRA with the accession code PRJNA768885 and PRJNA768942.

## References

[1] Martinez FJ, Collard HR, Pardo A, Raghu G, Richeldi L, Selman M (2017). Idiopathic pulmonary fibrosis. Nat Rev Dis. Prim.

[2] Jarzebska N, Karetnikova ES, Markov AG, Kasper M, Rodionov RN, Spieth PM (2020). Scarred lung. An update on radiation-induced pulmonary fibrosis. Front Med.

[3] Li N, Shi F, Wang X, Yang P, Sun K, Zhang L (2021). Silica dust exposure induces pulmonary fibrosis through autophagy signaling. Environ Toxicol..

[4] Zou JN, Sun L, Wang BR, Zou Y, Xu S, Ding YJ (2021). The characteristics and evolution of pulmonary fibrosis in COVID-19 patients as assessed by AI-assisted chest HRCT. PLoS ONE.

[5] He Y, Thummuri D, Zheng G, Okunieff P, Citrin DE, Vujaskovic Z (2019). Cellular senescence and radiation-induced pulmonary fibrosis. Transl Res.

[6] Epperly MW, Shields D, Fisher R, Hou W, Wang H, Hamade DF (2021). Radiation-induced senescence in p16+/LUC mouse lung compared to bone marrow multilineage hematopoietic progenitor cells. Radiat Res.

[7] Su L, Dong Y, Wang Y, Wang Y, Guan B, Lu Y (2021). Potential role of senescent macrophages in radiation-induced pulmonary fibrosis. Cell Death Dis..

[8] Sivananthan A, Shields D, Fisher R, Hou W, Zhang X, Franicola D (2019). Continuous 1 year oral administration of the radiation mitigator, MMS350, after total body irradiation restores bone marrow stromal cell proliferative capacity and reduces age-related senescence in Fanconi Anemia (Fanca-/-) mice. Radiat Res.

[9] Mora AL, Rojas M, Pardo A, Selman M (2017). Emerging therapies for idiopathic pulmonary fibrosis, a progressive age-related disease. Nat Rev Drug Discov.

[10] Herranz N, Gil J (2018). Mechanisms and functions of cellular senescence. J Clin Investig.

[11] Althubiti M, Lezina L, Carrera S, Jukes-Jones R, Giblett SM, Antonov A (2014). Characterization of novel markers of senescence and their prognostic potential in cancer. Cell Death Dis..

[12] Okuda R, Aoshiba K, Matsushima H, Ogura T, Okudela K, Ohashi K (2019). Cellular senescence and senescence-associated secretory phenotype: comparison of idiopathic pulmonary fibrosis, connective tissue disease-associated interstitial lung disease, and chronic obstructive pulmonary disease. J Thorac Dis..

[13] Chen Z, Cao K, Xia Y, Li Y, Hou Y, Wang L (2019). Cellular senescence in ionizing radiation (Review). Oncol Rep..

[14] Cisowski J, Sayin VI, Liu M, Karlsson C, Bergo MO (2016). Oncogene-induced senescence underlies the mutual exclusive nature of oncogenic KRAS and BRAF. Oncogene.

[15] Saleh T, Bloukh S, Carpenter VJ, Alwohoush E, Bakeer J, Darwish S (2020). Therapy-induced senescence: an “old” friend becomes the enemy. Cancers.

[16] Hernandez-Segura A, de Jong TV, Melov S, Guryev V, Campisi J, Demaria M (2017). Unmasking transcriptional heterogeneity in senescent cells. Curr Biol..

[17] Itahana K, Campisi J, Dimri GP (2007). Methods to detect biomarkers of cellular senescence: the senescence-associated beta-galactosidase assay. Methods Mol Biol..

[18] Sessions GA, Copp ME, Liu J-Y, Sinkler MA, D’Costa S, Diekman BO (2019). Controlled induction and targeted elimination of p16INK4a-expressing chondrocytes in cartilage explant culture. FASEB J..

[19] Weir MC, Shu ST, Patel RK, Hellwig S, Chen L, Tan L (2018). Selective inhibition of the myeloid Src-family kinase fgr potently suppresses AML cell growth in vitro and in vivo. ACS Chem Biol..

[20] Mirzayans R, Andrais B, Hansen G, Murray D (2012). Role of p16(INK4A) in replicative senescence and DNA damage-induced premature senescence in p53-deficient human cells. Biochem Res Int.

[21] Aranda-Anzaldo A (2009). A structural basis for cellular senescence. Aging.

[22] La Belle AA, Schiemann WP (2017). Oncostatin M activation of Stat3:Smad3 complexes drives senescence. Cell Cycle.

[23] Metais JY, Winkler T, Geyer JT, Calado RT, Aplan PD, Eckhaus MA (2012). Bcl2A1a over-expression in murine hematopoietic stem and progenitor cells decreases apoptosis and results in hematopoietic transformation. PLoS ONE.

[24] Moore BB, Murray L, Das A, Wilke CA, Herrygers AB, Toews GB (2006). The role of Ccl12 in the recruitment of fibrocytes and lung fibrosis. Am J Respir Cell Mol Biol..

[25] Zhang Q, Li YD, Zhang SX, Shi YY (2018). Centromere protein U promotes cell proliferation, migration and invasion involving Wnt/beta-catenin signaling pathway in non-small cell lung cancer. Eur Rev Med Pharm Sci..

[26] Avelar RA, Ortega JG, Tacutu R, Tyler EJ, Bennett D, Binetti P (2020). A multidimensional systems biology analysis of cellular senescence in aging and disease. Genome Biol..

[27] Kasmann L, Dietrich A, Staab-Weijnitz CA, Manapov F, Behr J, Rimner A (2020). Radiation-induced lung toxicity—cellular and molecular mechanisms of pathogenesis, management, and literature review. Radiat Oncol..

[28] Kalash R, Epperly MW, Goff J, Dixon T, Sprachman MM, Zhang X (2013). Amelioration of irradiation pulmonary fibrosis by a water-soluble bifunctional sulfoxide radiation mitigator (MMS350). Radiat Res.

[29] Li M, You L, Xue J, Lu Y (2018). Ionizing radiation-induced cellular senescence in normal, non-transformed cells and the involved DNA damage response: a mini review. Front Pharm..

[30] Acin-Perez R, Iborra S, Marti-Mateos Y, Cook ECL, Conde-Garrosa R, Petcherski A (2020). Fgr kinase is required for proinflammatory macrophage activation during diet-induced obesity. Nat Metab..

[31] Yilmaz O, Oztay F, Kayalar O (2015). Dasatinib attenuated bleomycin-induced pulmonary fibrosis in mice. Growth Factors.

[32] Ang X, Cai Y, Zhang W, Chen X (2018). Quercetin ameliorates pulmonary fibrosis by inhibiting SphK1/S1P signaling. Biochem Cell Biol..

[33] Xu M, Pirtskhalava T, Farr JN, Weigand BM, Palmer AK, Weivoda MM (2018). Senolytics improve physical function and increase lifespan in old age. Nat Med.

[34] Van Deursen JM (2014). The role of senescent cells in ageing. Nature.

[35] Mazzi P, Caveggion E, Lapinet-Vera JA, Lowell CA, Berton G (2015). The Src-family kinases Hck and Fgr regulate early lipopolysaccharide-induced myeloid cell recruitment into the lung and their ability to secrete chemokines. J Immunol..

[36] Davalli P, Mitic T, Caporali A, Lauriola A, D’Arca D (2016). ROS, cell senescence, and novel molecular mechanisms in aging and age-related diseases. Oxid Med Cell Longev..

[37] Acin-Perez R, Carrascoso I, Baixauli F, Roche-Molina M, Latorre-Pellicer A, Fernandez-Silva P (2014). ROS-triggered phosphorylation of complex II by Fgr kinase regulates cellular adaptation to fuel use. Cell Metab..

[38] Livak KJ, Schmittgen TD (2001). Analysis of relative gene expression data using real-time quantitative PCR and the 2−ΔΔCT method. Methods.

[39] Kasmann L, Dietrich A, Staab-Weijnitz CA, Manapov F, Behr J, Rimner A (2020). Radiation-induced lung toxicity—cellular and molecular mechanisms of pathogenesis, management, and literature review. Radiat Oncol..

[40] Su L, Dong Y, Wang Y, Wang Y, Guan B, Lu Y (2021). Potential role of senescent macrophages in radiation-induced pulmonary fibrosis. Cell Death Dis..

[41] Adams TS, Schupp JC, Poli S, Ayaub EA, Neumark N, Ahangari F (2020). Single-cell RNA-seq reveals ectopic and aberrant lung-resident cell populations in idiopathic pulmonary fibrosis. Sci Adv..

[42] Justice JN, Nambiar AM, Tchkonia T, LeBrasseur NK, Pascual R, Hashmi SK (2019). Senolytics in idiopathic pulmonary fibrosis: results from a first-in-human, open-label, pilot study. eBio Med..

[43] Wilkinson HN, Hardman MJ (2020). Senescence in wound repair: emerging strategies to target chronic healing wounds. Front Cell Dev Biol..

[44] Amor C, Feucht J, Leibold J, Ho YJ, Zhu C, Alonso-Curbelo D (2020). Senolytic CAR T cells reverse senescence-associated pathologies. Nature.

[45] Correia-Melo C, Hewitt G, Passos JF (2014). Telomeres, oxidative stress and inflammatory factors: partners in cellular senescence?. Longev Healthspan.

[46] Ferreira-Gonzalez S, Lu WY, Raven A, Dwyer B, Man TY, O’Duibhir E (2018). Paracrine cellular senescence exacerbates biliary injury and impairs regeneration. Nat Commun..

[47] Bird TG, Muller M, Boulter L, Vincent DF, Ridgway RA, Lopez-Guadamillas E (2018). TGF-beta inhibition restores a regenerative response in acute liver injury by suppressing paracrine senescence. Sci Transl Med.

[48] Kirkland JL, Tchkonia T (2020). Senolytic drugs: from discovery to translation. J Intern Med.

[49] Cazzola M, Matera MG, Rogliani P, Calzetta L (2018). Senolytic drugs in respiratory medicine: is it an appropriate therapeutic approach?. Expert Opin Investig Drugs.

[50] Zhu Y, Tchkonia T, Pirtskhalava T, Gower AC, Ding H, Giorgadze N (2015). The achilles’ heel of senescent cells: from transcriptome to senolytic drugs. Aging Cell.

[51] Vitali R, Mancini C, Cesi V, Tanno B, Piscitelli M, Mancuso M (2009). Activity of tyrosine kinase inhibitor Dasatinib in neuroblastoma cells in vitro and in orthotopic mouse model. Int J Cancer.

[52] Kirkland JL, Tchkonia T (2021). Senolytic drugs: from discovery to translation. J Intern Med.

[53] Yousefzadeh MJ, Zhu Y, McGowan SJ, Angelini L, Fuhrmann-Stroissnigg H, Xu M (2018). Fisetin is a senotherapeutic that extends health and lifespan. EBioMedicine.

[54] Schosserer M, Grillari J, Breitenbach M (2017). The dual role of cellular senescence in developing tumors and their response to cancer therapy. Front Oncol..

[55] Wilkinson HN, Hardman MJ (2020). Senescence in wound repair: emerging strategies to target chronic healing wounds. Front Cell Dev Biol..

[56] Yun MH (2018). Cellular senescence in tissue repair: every cloud has a silver lining. Int J Dev Biol..

[57] Hewitt RJ, Maher TM (2019). Idiopathic pulmonary fibrosis: new and emerging treatment options. Drugs Aging.

[58] Paliogiannis P, Fois SS, Fois AG, Cossu A, Palmieri G, Pintus G (2021). Repurposing anticancer drugs for the treatment of idiopathic pulmonary fibrosis and antifibrotic drugs for the treatment of cancer: state of the art. Curr Med Chem..

[59] Stahnke T, Kowtharapu BS, Stachs O, Schmitz KP, Wurm J, Wree A (2017). Suppression of TGF-beta pathway by pirfenidone decreases extracellular matrix deposition in ocular fibroblasts in vitro. PLoS ONE.

[60] Sato S, Shinohara S, Hayashi S, Morizumi S, Abe S, Okazaki H (2017). Anti-fibrotic efficacy of nintedanib in pulmonary fibrosis via the inhibition of fibrocyte activity. Respir Res.

[61] Fukihara J, Kondoh Y (2016). Nintedanib (OFEV) in the treatment of idiopathic pulmonary fibrosis. Expert Rev Respir Med.

[62] Behr J, Prasse A, Kreuter M, Johow J, Rabe KF, Bonella F (2021). Pirfenidone in patients with progressive fibrotic interstitial lung diseases other than idiopathic pulmonary fibrosis (RELIEF): a double-blind, randomised, placebo-controlled, phase 2b trial. Lancet. Respir Med.

[63] Greenberger JS (1978). Sensitivity of corticosteroid-dependent, insulin-resistant lipogenesis in marrow preadipocytes of mutation diabetic-obese mice. Nature.

[64] Sakakeeny MA, Greenberger JS (1982). Granulopoiesis longevity in continuous bone marrow cultures and factor dependent cell line generation: significant variation among 28 inbred mouse strains and outbred stocks. J Natl Cancer Inst..

[65] Berhane H, Epperly MW, Goff J, Kalash R, Cao S, Franicola D (2014). Radiobiologie differences between bone marrow stromal and hematopoietic progenitor cell lines from Fanconi Anemia (Fancd2−/−) mice. Radiat Res.

[66] Ejaz A, Epperly MW, Greenberger JS, Rubin PJ (2019). Adipose-derived stem cell therapy ameliorates ionizing irradiation fibrosis (RIF) via hepatocyte growth factor mediated TGF-β down regulation and recruitment of bone marrow cells. Stem Cells.

[67] Bao R, Stapor D, Luke JJ (2020). Molecular correlates and therapeutic targets in T cell-inflamed versus non-T cell-inflamed tumors across cancer types. Genome Med.

[68] Andrews S, Fast QC. A quality control application for high throughput sequence data. Babraham Institute. 2016; http://www.bioinformatics.babraham.ac.uk/projects/fastqc.

[69] Bray NL, Pimentel H, Melsted P, Pachter L (2016). Near-optimal probabilistic RNA-seq quantification. Nat Biotechnol..

[70] Soneson C, Love MI, Robinson MD (2015). Differential analyses for RNA-seq: transcript-level estimates improve gene-level inferences. F1000Res.

[71] Law CW, Chen Y, Shi W, Smyth GK (2014). voom: Precision weights unlock linear model analysis tools for RNA-seq read counts. Genome Biol..

[72] Angelidis I, Simon LM, Fernandez IE, Strunz M, Mayr CH, Greiffo FR (2019). An atlas of the aging lung mapped by single cell transcriptomics and deep tissue proteomics. Nat Commun..

[73] Zheng GX, Terry JM, Belgrader P, Ryvkin P, Bent ZW, Wilson R (2017). Massively parallel digital transcriptional profiling of single cells. Nat Commun..

[74] Tabib T, Morse C, Wang T, Chen W, Lafyatis R (2018). SFRP2/DPP4 and FMO1/LSP1 define major fibroblast populations in human skin. J Invest Dermatol.

[75] Lun ATL, Riesenfeld S, Andrews T, Dao TP, Gomes T, Marioni JC (2019). EmptyDrops: distinguishing cells from empty droplets in droplet-based single-cell RNA sequencing data. Genome Biol..

[76] Becht E, McInnes L, Healy J, Dutertre CA, Kwok IWH, Ng LG (2019). Dimensionality reduction for visualizing single-cell data using UMAP. Nat Biotechnol..

[77] Aran D, Looney AP, Liu L, Wu E, Fong V, Hsu A (2019). Reference-based analysis of lung single-cell sequencing reveals a transitional profibrotic macrophage. Nat Immunol..

