# Supplementary material for: Ionizing irradiation-induced Fgr in senescent cells mediates fibrosis
Source: Cell Death Discov. 2021 Nov 12;7:349. doi: 10.1038/s41420-021-00741-4 (PMC8585734; doi:10.1038/s41420-021-00741-4)
Supplement: Supplementary file 1 — Supplemental material [file 41420_2021_741_MOESM1_ESM.pdf]

Supplemental Figure 1

A

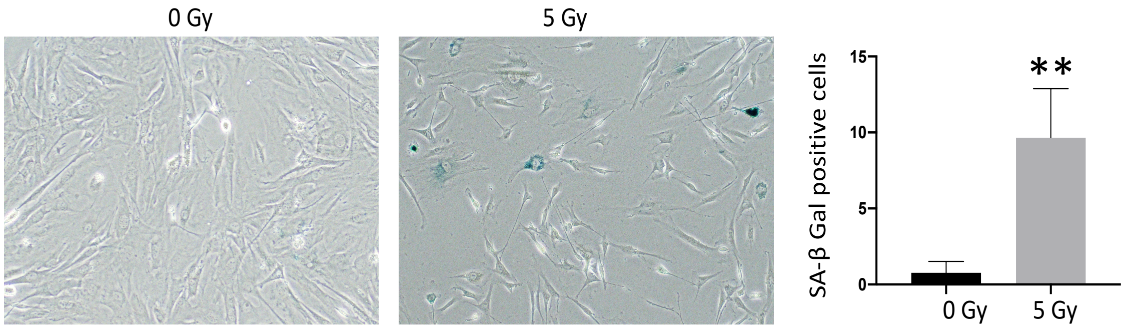

B

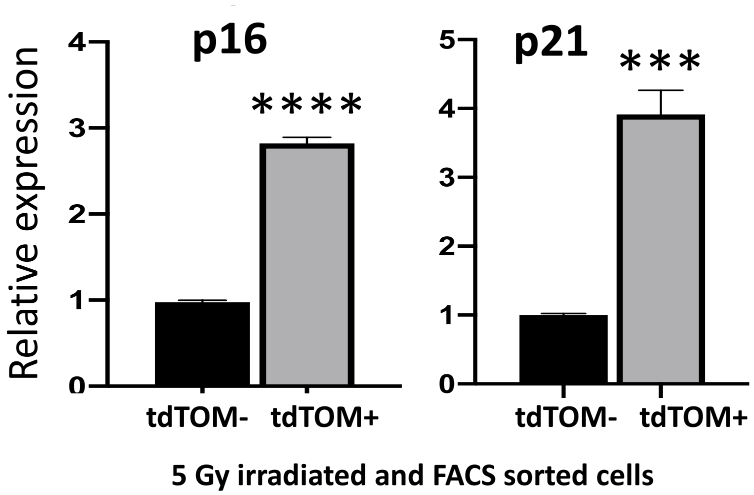

Supplemental Figure 1: Irradiation-induced tdTOMp16+ senescent cells display common biomarkers of senescence. A) SA-beta-gal staining after 10 days after 0 and 5 Gy irradiation in tdTOMp16+ cells. **B)** Genetic markers (p16 and p21) after FACS sorting by RT-qPCR. (n=3, **, p=< 0.0; ***, p=< 0.001)

Supplemental Figure. 2

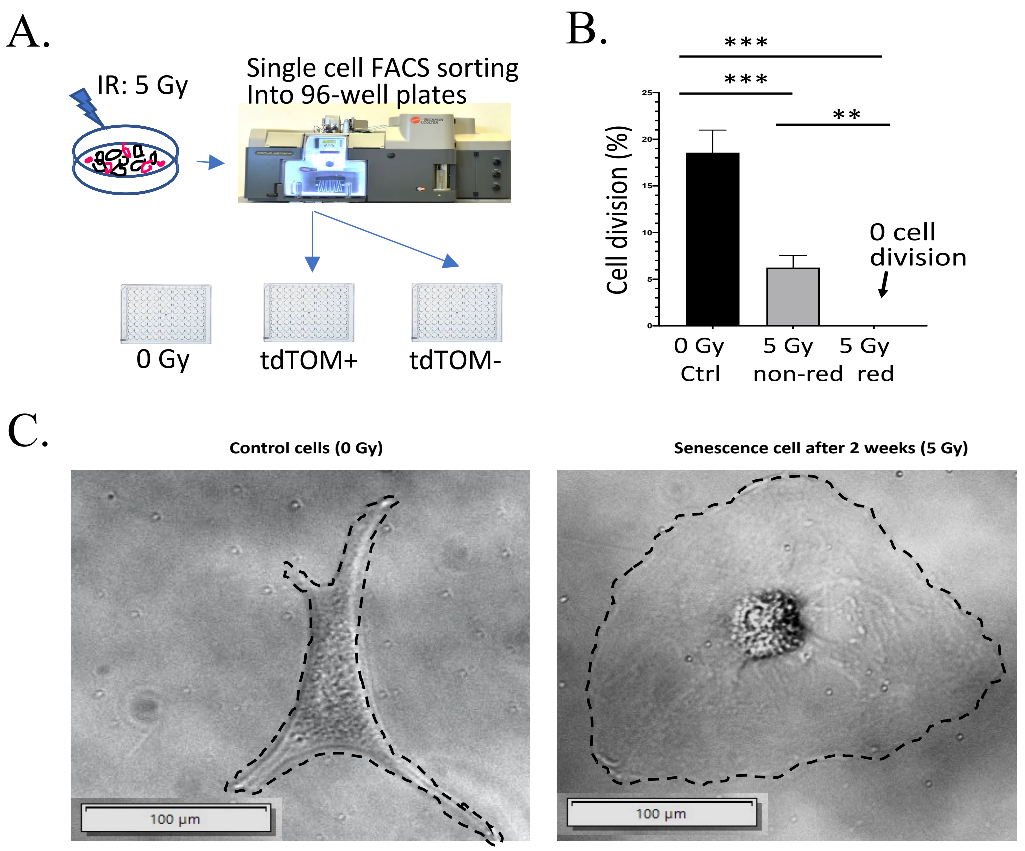

Supplemental Figure 2. Irradiation-induced and sorted tdTOMp16+ senescent cells are non-dividing. A) Single cells in 96 well plates. **B)** tdTOM+, 5 Gy, tdTOM- and non-irradiated cells 10 days (% cell division). **C)** Single non-irradiated cell (left), single larger, circular irradiated tdTOM+ sorted cell (right) after 2 weeks. (n=600-1000; *, p=< 0.5; **, p=<0.01)

Supplemental Figure 3

A.

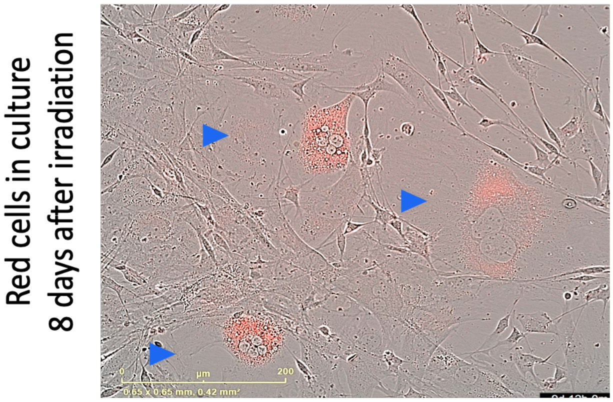

B.

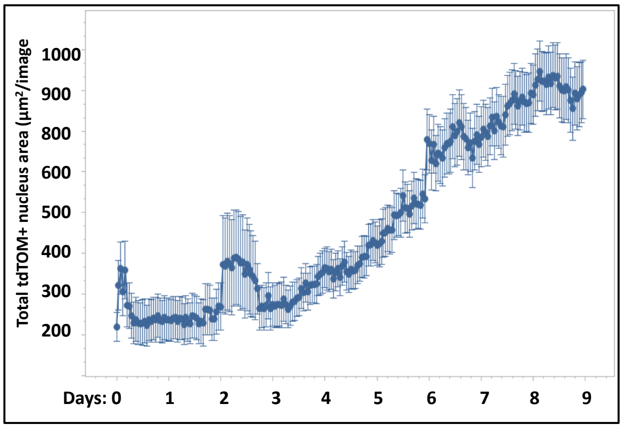

C. Movie

Supplemental Figure 3. Real time imaging of senescence. tdTOMp16+ cells irradiated (5 Gy), imaged hourly for 9 days **A)** tdTOM+ cells bigger, (arrowheads) surrounded by non-senescent cells. **B)** Senescent cells over 9 days. (IncuCyte® Live-Cell Analysis Systems from Essen BioScience, MI, USA, Lanigan TM, et al.) **C)** Movie.

Supplemental Figure 4

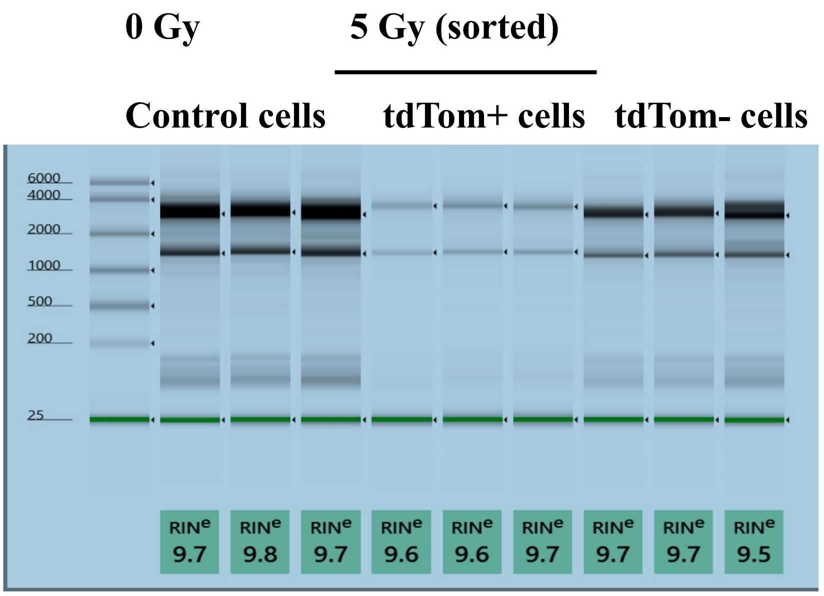

Supplemental Figure 4. RNA integrity of control cells and 5 Gy irradiated TOM+ cells and TOM- cells.

Supplemental Figure 5

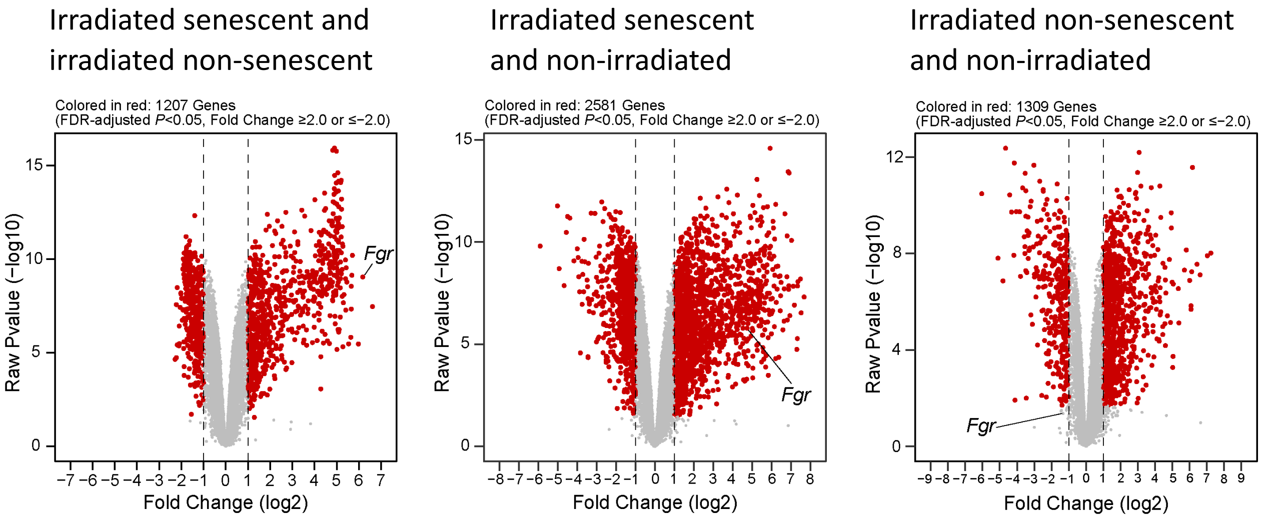

Supplemental Figure 9. Volcano plots showing Fgr expression when radiated senescent, radiated non-senescent, and non-irradiated cells are compared to each other in each pair. The plots show the log2 (fold change) versus the −log10 (adjusted p-value) for all of genes detected in the RNA-seq analysis (i.e., for both DEGs and non-DEGs). The grey color represents genes with no significantly different expression while the red color indicated over expression or under expression. Place of Fgr is shown by an arrow in each of the three comparisons.

Supplemental Figure 6

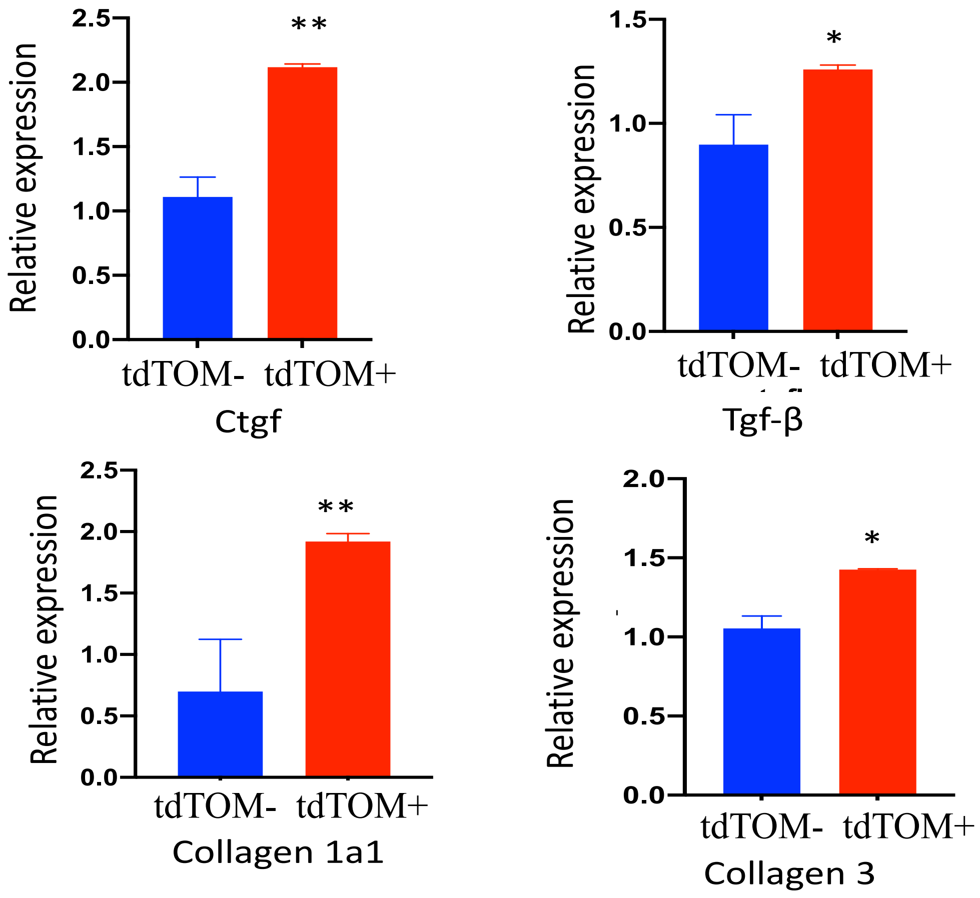

Supplemental Figure 5: Senescence biomarkers in tdTOMp16+ sorted cells from freshly explanted mouse tail skin fibroblasts. Irradiated (R) (5 Gy) and non-irradiated (NR) tdTOMp16+ cells on top and target fibroblasts on bottom. Target cells 10 days harvested for RNA and RT-qPCR for Ctgf, TGF-β, Collagen1a1, Collagen 3. (n=3, *, p=<0.05; **, p=0.01, NS=non-significant)

Supplemental Figure 7:

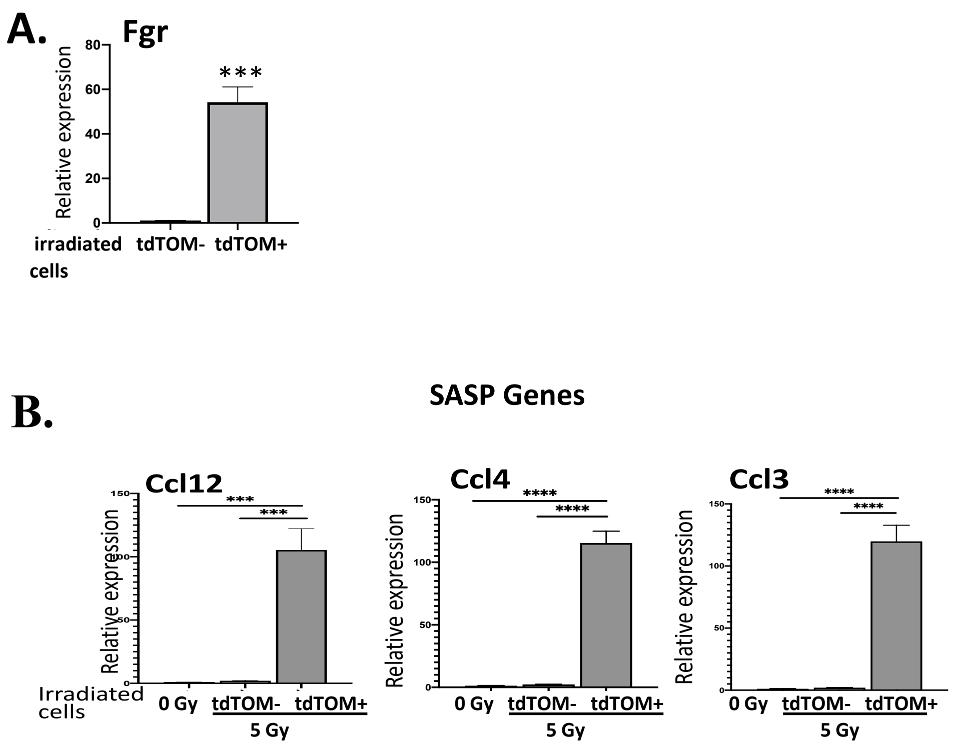

Supplemental Figure 6: Confirmation of increased abundance of specific transcripts by RT-qPCR. A) Relative expression of Fgr by RT-qPCR. **B)** SASP genes Ccl4, Ccl3, Ccl12 by RT-qPCR and RNA-seq. Error bars represent ± s.d. Overall, the trend of gene expression by RT-qPCR mirrors that of the RNA-seq analysis. (n=3; **, p=<0.01; ***, p=<0.001).

Supplemental Figure 8:

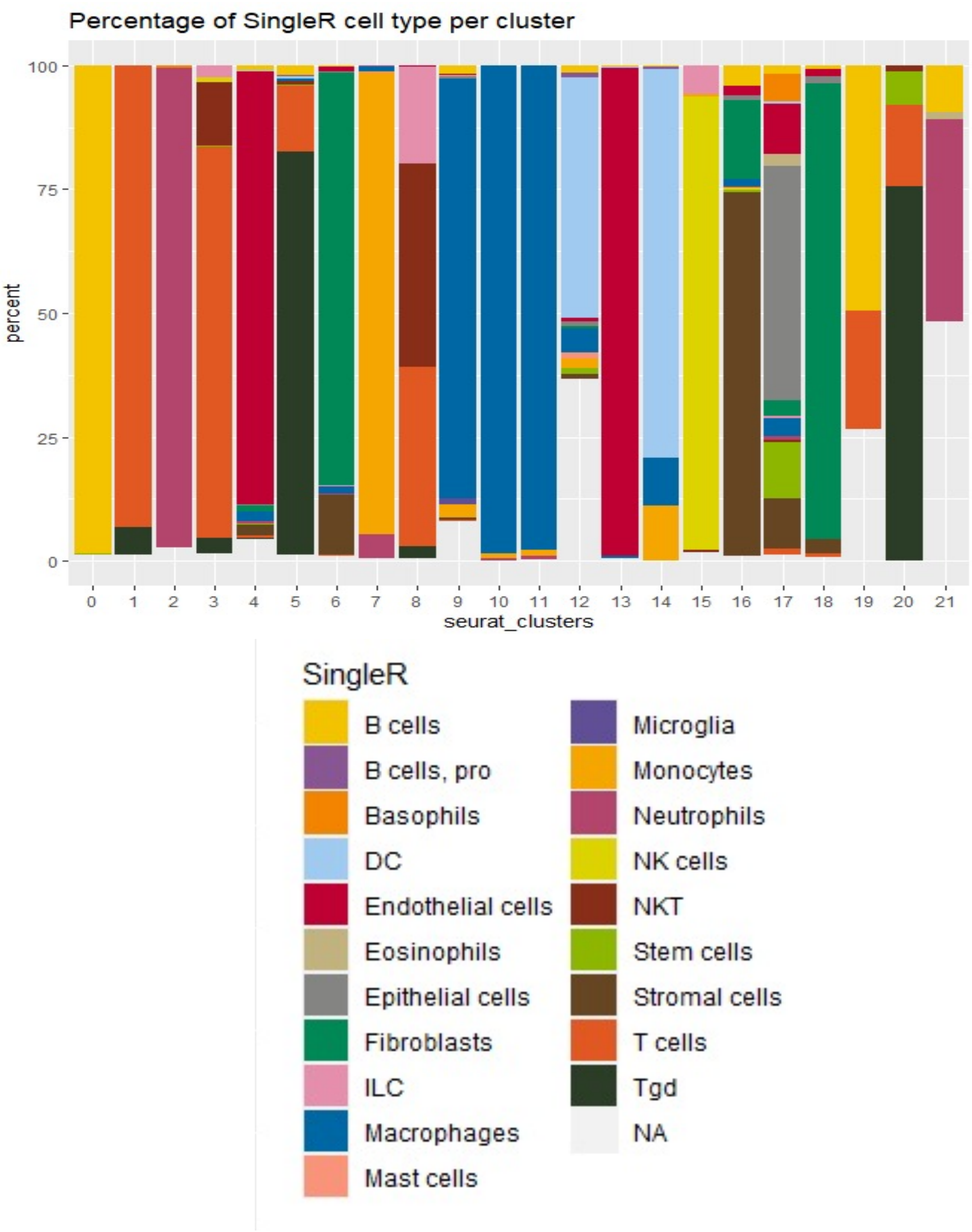

Supplemental Figure 7: Clusters of populations with proportion of cells in each cluster on UMAP. Single-cell RNA-seq was performed on single-cell suspensions generated mice with control and pulmonary fibrosis. All cells were used to generate 21 different clusters and their assigned cell phenotypes.

Supplemental Figure 9

	Gene names	Symbol
1	interleukin 6	IL6
2	interleukin 7	IL7
3	interleukin 15	IL15
4	C-X-C motif chemokine ligand 8	CXCL8
5	C-X-C motif chemokine ligand 1	CXCL1
6	C-X-C motif chemokine ligand 2	CXCL2
7	C-C motif chemokine ligand 8	CCL8
8	C-C motif chemokine ligand 13	CCL13
9	C-C motif chemokine ligand 3	CCL3
10	C-C motif chemokine ligand 20	CCL20
11	matrix metallopeptidase 1	MMP1
12	matrix metallopeptidase 3	MMP3
13	matrix metallopeptidase 10	MMP10
14	matrix metallopeptidase 12	MMP12
15	matrix metallopeptidase 13	MMP13
16	matrix metallopeptidase 14	MMP14
17	TIMP metallopeptidase inhibitor 1	TIMP1
18	TIMP metallopeptidase inhibitor 2	TIMP2
19	serpin family E member 1	SERPINE1
20	serpin family B member 2	SERPINB2
21	plasminogen activator, tissue type	PLAT
22	plasminogen activator, urokinase	PLAU
23	cathepsin B	CTSB

Supplemental Figure 8: SASP genes in senescent cells in mouse lungs by scRNAseq. 23 major genes were shortlisted and analyzed for induction of senescence in different cell types identified by scRNAseq in control and RIPF mouse lungs. 23 genes were upregulated in every senescent cell.

Supplemental Figure 10

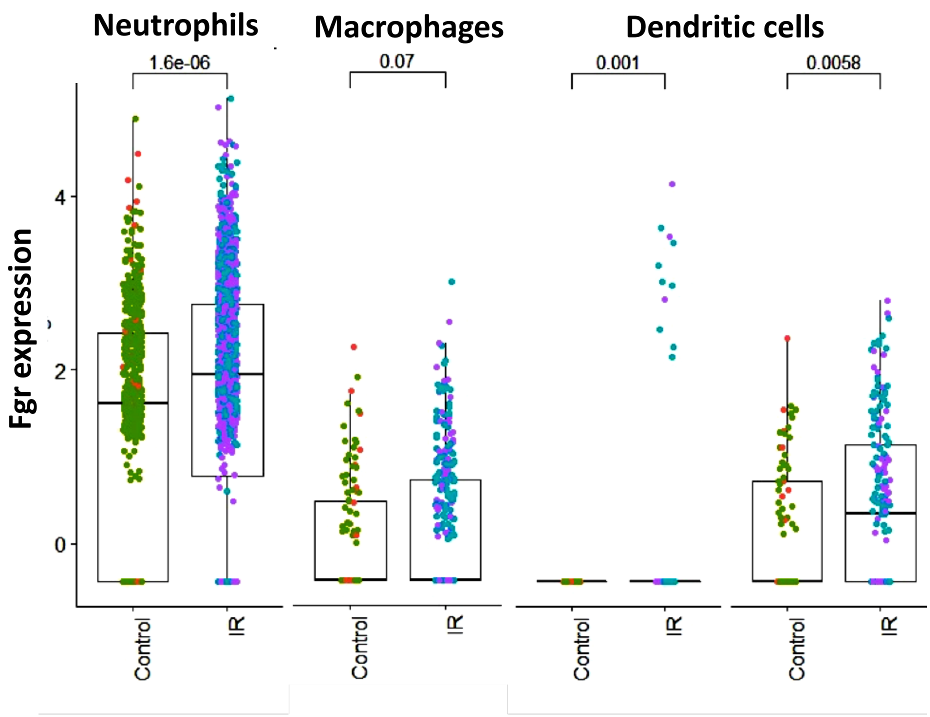

Supplemental Figure 10. Fgr expression in RIPF lung cells at day 150 after 20 Gy thoracic irradiation compared to control lungs. scRNAseq analysis of Fgr expressing cell types shows upregulation of Fgr in neutrophils (p=1.6e-06), macrophages (p=0.07) and dendritic cells (two clusters, p=0.001 and p=0.0058).
